# The role of Q10 engineering mesenchymal stem cell-derived exosomes in inhibiting ferroptosis for diabetic wound healing

**DOI:** 10.1093/burnst/tkae054

**Published:** 2024-11-26

**Authors:** Ronghua Yang, Sitong Zhou, Jie Huang, Deni Kang, Yao Chen, Xinyi Wang, Yan Shi, Zhengguang Wang

**Affiliations:** Department of Burn and Plastic Surgery, Guangzhou First People's Hospital, Guangzhou Medical University, South China University of Technology, Panfu Road, Yuexiu District, Guangzhou, Guangdong, 510180, China; Department of Dermatology, The First People’s Hospital of Foshan, Lingnan North Road, Chancheng District, Foshan, Guangzhou, 528000, China; Department of Burn and Plastic Surgery, Guangzhou First People's Hospital, Guangzhou Medical University, South China University of Technology, Panfu Road, Yuexiu District, Guangzhou, Guangdong, 510180, China; Department of Burn and Plastic Surgery, Guangzhou First People's Hospital, Guangzhou Medical University, South China University of Technology, Panfu Road, Yuexiu District, Guangzhou, Guangdong, 510180, China; Department of Burn and Plastic Surgery, Guangzhou First People's Hospital, Guangzhou Medical University, South China University of Technology, Panfu Road, Yuexiu District, Guangzhou, Guangdong, 510180, China; Department of Burn and Plastic Surgery, Guangzhou First People's Hospital, Guangzhou Medical University, South China University of Technology, Panfu Road, Yuexiu District, Guangzhou, Guangdong, 510180, China; Department of Plastic, Medical Center of Burn Plastic and Wound Repair, The First Affiliated Hospital of Nanchang University, Jiangxi Medical College, Nanchang University, Yongwaizheng Road, Donghu District, Nanchang, Jiangxi 330006, China; Department of Orthopaedics, Peking University Third Hospital, 49 North Garden Road, Haidian District, Beijing, 100191, China

**Keywords:** Ferroptosis, Exosomes, Coenzyme Q10, ACSL4, Diabetic wound healing, Keratinocytes, Mesenchymal stem cells, Keratinocyte

## Abstract

**Background:**

Ferroptosis plays an essential role in the development of diabetes and its complications, suggesting its potential as a therapeutic target. Stem cell-derived extracellular vesicles (EVs) are increasingly being developed as nano-scale drug carriers. The aim of this study was to determine the role of ferroptosis in the pathogenesis of diabetic wound healing and evaluate the therapeutic effects of coenzyme Q10 (Q10)-stimulated exosmes derived from mesenchymal stem cells (MSCs).

**Methods:**

Human keratinocytes (HaCaTs) were exposed to high glucose (HG) conditions *in vitro* to mimic diabetic conditions, and the ferroptosis markers and expression level of acyl-coenzyme A synthase long-chain family member 4 (ACSL4) were determined. Exosomes were isolated from control and Q10-primed umbilical cord mesenchymal stem cells (huMSCs) and characterized by tramsmission electron microscopy and immunofluorescence staining. The HG-treated HaCaTs were cultured in the presence of exosomes derived from Q10-treated huMSCs (Q10-Exo) and their *in vitro* migratory capacity was analyzed.

**Results:**

Q10-Exo significantly improved keratinocyte viability and inhibited ferroptosis *in vitro*. miR-548ai and miR-660 were upregulated in the Q10-Exo and taken up by HaCaT cells. Furthermore, miR-548ai and miR-660 mimics downregulated ACSL4-inhibited ferroptosis in the HG-treated HaCaT cells and enhanced their proliferation and migration. However, simultaneous upregulation of ACSL4 reversed their effects. Q10-Exo also accelerated diabetic wound healing in a mouse model by inhibiting ACSL4-induced ferroptosis.

**Conclusions:**

Q10-Exo promoted the proliferation and migration of keratinocytes and inhibited ferroptosis under hyperglycemic conditions by delivering miR-548ai and miR-660. Q10-Exo also enhanced cutaneous wound healing in diabetic mice by repressing ACSL4-mediated ferroptosis.

## Graphical Abstract

**Graphical Abstract:**
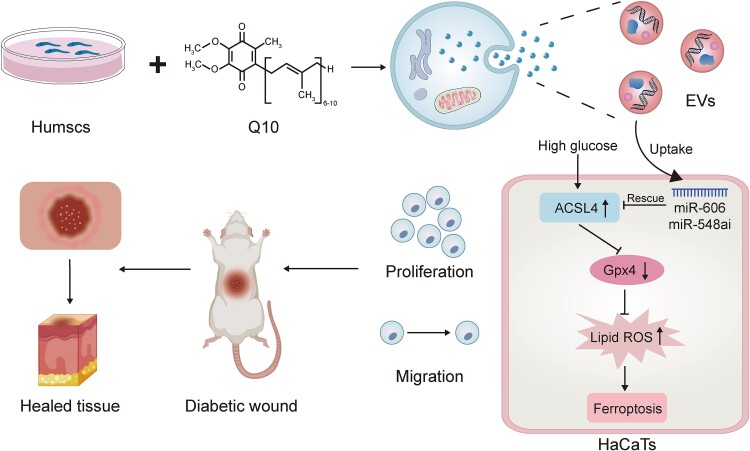

Highlights

- The study describes for the first time that Q10-Exo significantly improved keratinocyte viability and inhibited the ferroptosis process *in vitro*.
- The study demonstrated that miR-548ai and miR-660 mimics downregulated ACSL4-inhibited ferroptosis in HG-treated HaCaT cells and enhanced their proliferation and migration
- Q10-Exo also accelerated diabetic wound healing in a mouse model by inhibiting ACSL4-induced ferroptosis.

## Background

Almost 25% of patients with diabetes eventually develop diabetic foot ulcers [1]. Although numerous therapeutic approaches have been devised for chronic wounds, the cure ratio remains low and ~ 28% of the patients require lower limb amputation [2–4]. Diabetic conditions delay wound healing due to the disruption in glucose homeostasis and elevated glucose levels [5, 6].

Wound healing is a dynamic process consisting of four sequential and overlapping phases, namely hemostasis, inflammation, re-epithelialization, and tissue remodeling [7]. Re-epithelialization occurs during the proliferative phase of wound healing and is characterized by the migration and proliferation of keratinocytes into the wound bed. Ineffective and delayed wound healing in diabetic patients is partially caused by dysfunctional epidermal keratinocytes [8, 9] in response to hyperglycemia [10]. Furthermore, keratinocytes and inflammatory cells produce high levels of reactive oxygen species under hyperglycemic conditions, resulting in tissue damage during diabetic wound healing [11–13].

Ferroptosis is an iron-dependent form of programmed cell death that is accompanied by the accumulation of lipid peroxides [14]. Long-lasting hyperglycemia triggers excessive lipid peroxidation, which in turn impairs iron metabolism [15]. In this study, we found that high glucose (HG) conditions induced ferroptosis in cultured keratinocytes, and the deleterious effects of hyperglycemia were significantly reduced by blocking the ferroptosis pathway. This is consistent with recent studies that have demonstrated that inhibition of ferroptosis can accelerate diabetic wound healing.

Exosomes isolated from specific cell types can facilitate the healing of diabetic wounds through their cargoes consisting of nucleic acids, proteins, and lipids [16]. For instance, exosomal miR-20b-5p from diabetic patients accelerated wound repair by promoting angiogenesis [17]. In addition, exosomes secreted from umbilical cord mesenchymal stem cells (huMSCs) can mediate regenerative wound healing [18–21] through various paracrine factors [20, 22, 23]. However, exosomes derived from stem cells may induce senescence in the recipient cells, and even undergo cargo modification [24]. Studies show that preconditioning stem cells with drugs may increase the production of exosomes and enrich the bioactive cargoes, eventually enhancing their therapeutic potential [25, 26]. Pre-treatment with different drugs has been shown to alter the content of microRNAs (miRNAs), mRNAs, lipids, and proteins in the exosomes secreted by stem cells [27]. Several exosomal miRNAs have been identified in recent years that exert therapeutic effects by regulating the expression of target mRNAs [28, 29]. For instance, adipose-derived stem cell exosomes expressing high levels of miR-21 enhance the migration and proliferation of human keratinocytes (HaCaT cells) by upregulating Matrix metalloproteinase-9 (MMP-9), thereby promoting wound healing [30]. In addition, bone marrow-derived stem cells (BMSCs) pre-treated with desferrioxamine (DFO) secrete exosomes overexpressing miR-126, which promote diabetic wound healing by enhancing angiogenesis through the activation of endothelial cells [31]. Likewise, exosomes derived from atorvastatin-treated MSCs alleviated myocardial infarction in a rat model by inhibiting inflammation and promoting angiogenesis through Long non-coding RNA (lncRNA) H19 [32]. Fisetin treatment increased Telomerase reverse transcriptase (TERT) expression in keratinocyte-derived exosomes, which induced proliferation of hair follicle stem cells by increasing β-linked proteins and mitochondria [33]. Furthermore, exosomes derived from metformin-treated MSCs mitigated disc cell senescence *in vitro* and *in vivo* [34].

Coenzyme Q10 (Q10) is a vitamin-like, oil-soluble molecule that is present at the mitochondrial inner membrane in exceptionally high concentrations [35]. It is widely used as a dietary supplement and can significantly improve or reverse stem-cell aging. However, it is not known whether Q10 pretreatment of huMSC-exosomes is capable of reversing HG-induced keratinocyte impairment and ferroptosis. This study aimed to evaluate the therapeutic effects of exosomes derived from Q10-treated huMSCs (Q10-Exo) on diabetic wound healing using *in vitro* and *in vivo* models, and explore the exosomal miRNAs [21, 36]. We found that Q10-Exo promoted diabetic wound healing by inhibiting acyl-coenzyme A synthase long-chain family member 4 (ACSL4) and suppressing ferroptosis, and identified two exosomal miRNAs as the therapeutic mediators. Therefore, Q10-Exo is a promising therapeutic option for accelerating wound healing in diabetic patients.

## Methods

### Keratinocyte culture and treatment

HaCaT were procured from Procell Life Science & Technology (CL-0090) and maintained in minimum essential medium (Gibco, A3160802) supplemented with 10% fetal bovine serum. The cells were incubated at 37°C in a humid environment with 95% air and 5% CO_2_. To simulate the HG microenvironment of a diabetic wound, the HaCaT cells were cultured in the presence of 50 mM glucose. The control cells were cultured in normoglycemic (5.5 mM glucose) Dulbecco's Modified Eagle Medium (DMEM) supplemented with 10% fetal bovine serum and 1% antibiotic–antimycotic solution for 48 h [10]. As per the experimental requirements, the cells (5 × 10^6^) were further treated with BMSC-Exos (100 μg/ml), Q10-BMSC-Exos (100 μg/ml) or phosphate buffered saline (PBS) (control) for 24 h.

### huMSCs culture and Q10 treatment

huMSCs were purchased from Guangdong VitaLife Biotechnology Co., Ltd (Foshan, China). The cells were seeded in a 96-well plate at a density of 5 × 10^3^ cells/well and treated with different concentrations of Q10 (0.2, 1, and 5 mM) or PBS (control) for 24 h. To assess viability, 10 μl of Cell Counting Kit-8 (CCK-8) solution was added to each well and the cells were incubated for 4 h. The absorbance of the samples was measured at 450 nm using a spectrophotometer (BioTek, Winooski, VT, USA). The viability rates in the experimental groups were calculated as the change in optical density (OD) relative to that in the control group (supplementary Fig. 1A, see online supplementary material). The 1 mM concentration was chosen as being optimal for subsequent treatments.

### Exosome extraction and identification

Exosomes were isolated from the control (Ctrl-Exo) and Q10-treated (Q10-Exo) huMSCs as described previously [2]. Briefly, the culture supernatant was collected after 48 h and centrifuged at 300 × g for 10 min and 3000 × g for 20 min to remove cell debris. The supernatant was filtered through a 0.22-μm pore-size membrane (Guangzhou Jet Bio-Filtration Co., Ltd), centrifuged at 10 000 × g for 30 min, and transferred to a new ultrafiltration tube. This supernatant was centrifuged at 110 000 × g for 70 min and the pellet was resuspended in PBS (Guangzhou Jet Bio-Filtration Co., Ltd) and centrifuged at 110 000 g for 70 min. The pellet was then resuspended in the appropriate volume of PBS for exosome characterization. All operations were performed at 4°C. The expression of CD9 (1 : 1000), CD63 (1 : 1000), and CD81 (1 : 1000) (Abcam, UK) was detected by western blotting to confirm that the exosome isolations were successful.

### Transmission electron microscopy

The suitably treated cells were fixed with 2.5% glutaraldehyde for 5 min at 25°C, scraped from the plates, and spun for 5 min at 500 g. Cell pellets were fixed in 2.5% glutaraldehyde in the dark at 25°C for 30 min. After embedding in 1% agarose, cells or tissues were post-fixed in 1% osmium tetroxide for 2 h, dehydrated with ethanol and acetone, and embedded in epoxy resin (SPI, 90, 529–77-4). The blocks were sliced into sections and stained with ethanolic uranyl acetate and lead citrate.

### Malondialdehyde assay

The malondialdehyde (MDA) content in the suitably treated cells was measured using a lipid peroxidation MDA assay kit (Beyotime, Jiangsu, China). The keratinocytes were seeded in a 6-well plate and treated as above for 4 h. The cells were homogenized in cell lysis buffer mixed with protease inhibitor to extract the protein fraction, and 100 μl of the lysates or standard solution were mixed with 200 μl of MDA detection working buffer. The samples were heated in a hot iron block at 100°C for 15 min and cooled to room temperature in a water bath. After centrifuging at 1000 x g for 10 min at room temperature, the absorbance of the samples was measured at 532 nm. The MDA concentrations were calculated relative to the total protein concentration. Proteins were quantified using a BCA protein assay kit (Thermo, Waltham, USA).

### *In vitro* wound-healing assay

The cells were seeded in 12-well plates and incubated for 2 h with 5 μg/ml mitomycin-C (S8146, Selleck). The monolayers were scratched using a 10-μl plastic pipette tip to create a ‘wound’, and the wells were washed with medium to remove any cell debris. An inverted light microscope was used to monitor the wound-healing process (Olympus, Japan). The migratory capacity of HaCaTs was determined by calculating the wound-closure rate (%) with ImageJ software.

### miRNA-sequencing analysis

Total RNA was extracted from the exosomes using a SeraMir exosome RNA kit (SBI, Mountain View, USA). RNA-seq was conducted on the Illumina HiSeq 2500 platform at Haplox (Guangzhou, China). Briefly, the RNAs were ligated with 3′ and 5′ adapters, and amplified by low-cycle RT-PCR. The amplicons were size-selected by Polyacrylamide gel electrophoresis (PAGE) gel according to the instructions of NEBNext® Multiplex Small RNA Library Prep Set for Illumina® (Illumina, USA). Before clustering, each library was diluted to a final concentration of 10 nM and equimolar amounts were pooled. To meet the sample compliance criteria, paired-end sequencing, gene coverage, and thermal graph analysis were performed. The pheatmap and ggplot2 package in R software were applied to draw the heat and volcano maps.

### RNA extraction and real-time PCR

Total RNA was isolated from the huMSCs and HaCaTs using RNAiso Plus (TaKaRa Biotechnology, Shiga, Japan) according to the manufacturer’s instructions, and reverse transcribed to cDNA. Real-time PCR was performed on an ABI Prism 7500 Sequence Detection System (ABI, CA, USA) using SYBR Premix ExTaq (TaKaRa Biotechnology). Primer sequences are listed in Table 1.

**Table 1:** miRNAs and mRNA primer sequences.

Gene	Sequence (5′ − 3′)
GAPDH forward	GGGAAACTGTGGCGTGAT
GAPDH reverse	GAGTGGGTGTCGCTGTTGA
hsa-U6-forward	CTCGCTTCGGCAGCACA
hsa-U6-reverse GPX4 forward	AACGCTTCACGAATTTGCGT TAAGAACGGCTGCGTGGT
GPX4 reverse	GTAGGGGCACACACTTGTAGG
ACSL4 forward	AACCCAGAAAACTTGGGCATT
ACSL4 reverse	GTCGGCCAGTAGAACCACT
SLC7A11 forward	AACCCAGAAAACTTGGGCATT
SLC7A11 reverse	ATACGCTGAGTGTGGTTTGC
hsa-miR-96-3p forward hsa-miR-96-3p reverse hsa-miR-148a-5p forward hsa-miR-148a-5p reverse hsa-miR-301b-3p forward hsa-miR-301b-3p reverse hsa-miR-548ai forward hsa-miR-548ai reverse hsa-miR-548ap-3p forward hsa-miR-548ap-3p reverse hsa-miR-606 forward hsa-miR-606 reverse hsa-miR-628-5p forward hsa-miR-628-5p reverse	CAATCATGTGCAGTGCCAATAT AGTGCAGGGTCCGAGGTATT GATAGAAGTCAGTGCACTACAG AGTGCAGGGTCCGAGGTATT GCAATGATATTGTCAAAGCATCTG AGTGCAGGGTCCGAGGTATT ATCACAATTACTTTTGCATCAACC AGTGCAGGGTCCGAGGTATT CCACAATTACTTTTTACTGACCTAAAGA AGTGCAGGGTCCGAGGTATT CTGAAAATCAAAGATACAAGTGCC AGTGCAGGGTCCGAGGTATT TGGCAGTCGAAGGGAAGG AGTGCAGGGTCCGAGGTATT

### ACSL4 overexpression in HaCaT cells

HaCaT cells were transduced with control (Ad-GFP) or ACSL4-overexpressing (Ad-ACSL4) adenoviruses at 10 Multiplicity of infection (MOI) (Research-Bio, Shanghai, China). The cells were also transfected with scrambled control oligonucleotides (50 nM; GenePharma, Shanghai, China) using Lipofectamine 3000 according to the manufacturer’s instructions.

### Establishment of an *in vivo* full-thickness cutaneous wound healing model

All animal experiments were conducted according to the guidelines of Shenzhen People’s Hospital for animal experiments, which met the National institutes of health (NIH) guidelines for the care and use of laboratory animals. WT and DB/DB male mice (7 weeks old) were purchased from Shanghai Slac Laboratory Animal Co. Ltd (Shanghai, China) and housed under a 12 h light/dark cycle at constant temperature (20°C). The full-thickness cutaneous wound model was developed as described previously [2]. Briefly, the mice were anesthetized with 5% pelltobarbitalum natricum (25 mg/kg) and sumianxin (0.1 ml/kg). A punch biopsy instrument was applied on the dorsal skin with moderate force to create a circular wound measuring 8 mm in diameter. The skin was sharply excised along the outline of the wound with a pair of scissors, leaving subcutaneous dorsal muscle exposed. The mice were euthanized 14 days after wounding, and the wound tissues were fixed and paraffin-embedded for hematoxylin–eosin (HE) and Masson staining.

### Luciferase reporter assay

To construct the luciferase reporter plasmids, 3′-Untranslated region (UTR) of ACSL4 with wild-type (WT) or mutated binding sites were cloned into the pMIR luciferase reporter plasmids of miR-548ai and miR-606 respectively. The cells were co-transfected with the reporter constructs (10 μg) along with miR-548ai and miR-606 mimics using Lipofectamine 3000. Luciferase activity was quantified 48 h later using a dual-luciferase reporter assay kit (Promega) according to the instructions. SpectraMax M5 software (Molecular Devices, CA, USA) was used to analyze the data.

### HE and Masson’s trichrome staining

Skin tissues were fixed with 4% paraformaldehyde, embedded in paraffin, and cut into thin sections. HE and Masson’s trichrome staining were performed according to the standard protocols. Epidermal thickness, the length and area of epithelization, and the extent of collagen deposition were analyzed using ImageJ software.

### Statistical analysis

Data are presented as means of at least three separate experiments. Graph PadPrism (GraphPad Software Inc; La Jolla, CA, USA) was used for all statistical analyses. The differences between two groups were compared by the Student’s t-test, and multiple groups were compared using one-way Analysis of variance (ANOVA) and Tukey’s multiple comparison test. *P* < 0.05 was considered statistically significant.

## Results

### Diabetic wound healing is accompanied by ferroptosis and downregulation of ACSL4

**Figure 1:**
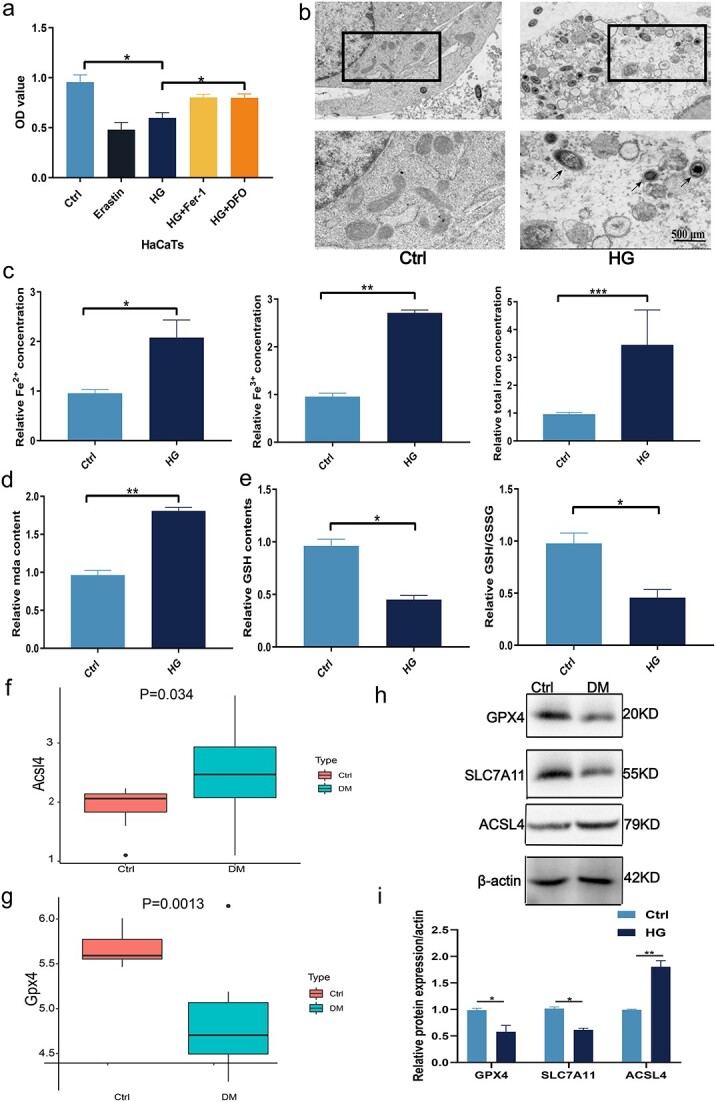
ACSL4 triggers ferroptosis in the wound healing process exposed to HG conditions. (**a**) CCK-8 assay was performed to assess the effect of HG treatment on HaCaTs viability with the other treatments. (**b**) Representative TEM images show mitochondrial membrane rupture of HaCaT cells in HG and control (Ctrl) groups. The lower panels show larger magnifications of the outlined boxes in the upper panels. Black arrows point to mitochondria. Scale bar: 500 nm. (**c**) Fe2^+^, Fe^3+^, and total iron content of HaCaT cells were measured in HG and control groups. (**d**) Relative MDA content was measured by the MDA detection kit in HaCaT cells in the HG and control groups. (**e**) GSH contents and the ratio of GSH/GSSG of HaCaT cells were measured in the HG and control groups. (**f**,**g**) Genome-wide RNA-Seq data of ACSL4 and GPX4 expression levels from wound healing tissues from control and diabetic (DM) rats on day 14. (**h**,**i**) Expression levels of GPX4, ACSL4, and SLC7A11 were analyzed by western blot from control and DM rats on day 14. ^*^p < 0.05, ^**^p < 0.01,^***^p < 0.001. *HG* high glucose, *MDA* malondialdehyde, *GSH* glutathione, *GSSG* glutathione disulfide

Based on previous reports, we treated HaCaT cells with 50 mM glucose (HG) to simulate the diabetic microenvironment [37]. The control cells were treated with 5 mM glucose [2, 10]. Both HG conditions and the ferroptosis inducer erastin signficantly reduced the viability of the HaCaT cells. In contrast, the iron chelator DFO and lipid peroxidation inhibitor ferrostatin-1 neutralized the effects of hyperglycemia (Figure 1a). This indicated that the HG-induced decrease in the viability of keratinocytes is mediated by ferroptosis. Mitochondrial disruption is a characteristic feature of ferroptosis. As shown in the transmission electron microscopy (TEM) images in Figure 1b, the mitochondrial cristae were reduced or disappeared, and the outer mitochondrial membrane was ruptured following HG exposure. To further confirm the role of ferroptosis in wound healing under chronic hyperglycemia, we induced full-thickness epidermal wounds in diabetic (DB/DB) and age-matched wild-type (WT) mice. As shown in Figure 1c, Fe^2+^, Fe^3+^, and total iron levels were significantly elevated in the skin tissues of DB/DB mice compared to that of control mice, indicating iron accumulation in diabetic skin wounds. Glutathione (GSH) depletion, and the accumulation of iron and MDA are the hallmarks of ferroptosis [38]. Therefore, to assess the extent of lipid peroxidation in skin tissues, we also measured the levels of MDA and GSH. As shown in Figure 1d, there was significant GSH depletion and MDA accumulation in the skin tissues of DB/DB mice, which was accompanied by a decrease in GSH/glutathione disulfide (GSSG) level. These findings confimed the occurrence of ferroptosis during diabetic skin wound healing.

Ferroptosis occurs due to a decrease in glutathione peroxidase (GPX4) activity, which leads to the accumulation of peroxides caused by the oxidation of polyunsaturated fatty acids. On the other hand, ACSL4 promotes ferroptosis by oxidizing cell membrane phospholipids [39]. Therefore, we next compared the expression of ferroptosis-related genes in the transcriptomic datasets of diabetic foot skin and acute wound skin tissues (GSE97615 and GSE134431) [40–42] (supplementary Fig. 1B). We utilized the non-parametric Wilcoxon signed-rank test to compare the two groups: ACSL4 was upregulated and GPX4 was downregulated in the diabetic skin tissues compared to the normal tissues (Figure 1f and g). Consistent with this, the anti-ferroptosis genes GPX4 and solute carrier family 7 member 11 (SLC7A11) were markedly downregulated during the diabetic wound healing process, and their expression levels were also significantly lower in the skin of DB/DB mice compared to the normal skin tissues. In contrast, ACSL4 was significantly upregulated in the diabetic skin tissues (Figure 1h and i).

### Characterization of huMSC-derived exosomes

**Figure 2:**
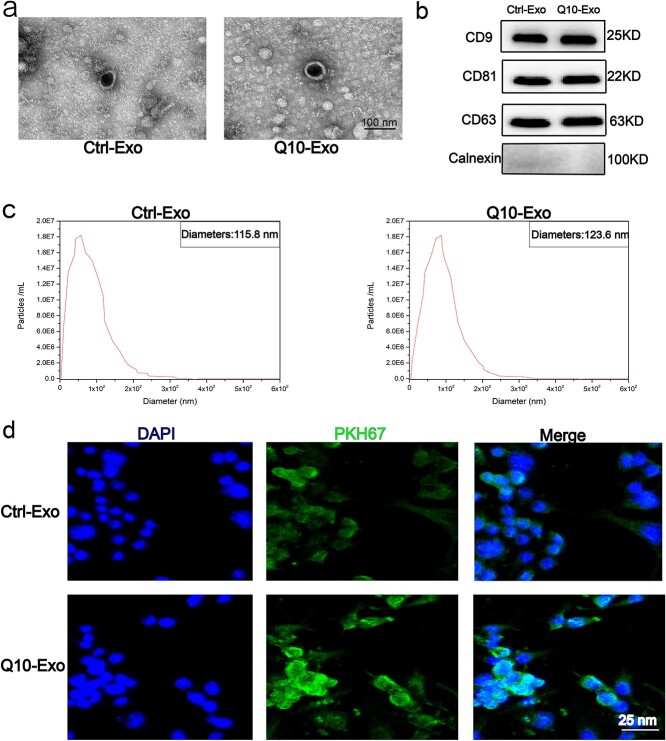
Characterization of exosomes derived from huMSCs. (**a**) TEM was utilized to visualize the morphology of exosomes and Q10-exo. The scale bar is 100 nm. (**b**) Western blotting of marker proteins (CD9, CD81, CD63, and calnexin) in Ctrl-Exo and Q10-Exo. (**c**) Nanoparticle tracking analysis was utilized to determine the particle size and concentrations of exosomes and Q10-Exo. (**d**) Uptake of PKH67-labeled exosomes and Q10-Exo by HaCaTs was studied using laser scanning confocal microscopy. The exosomes and the nucleus are each stained by PKH67 and DAPI.scale bar: 25 μm. *Ctrl* control, *EV* extracellular vesicle

Exosomes were isolated from the supernatant of Ctrl-Exo and Q10-Exo huMSCs, and characterized by TEM, western blotting, and nanoparticle tracking analysis. The morphology of the exosomes is shown in the TEM images in Figure 2a. The expression levels of CD9, CD63, and CD81 were similar in the Ctrl-Exo and Q10-Exo groups (Figure 2b). Interestingly, the Q10-Exo group showed a larger size distribution (average 123.6 nm) compared to the Ctrl-Exo group (average 115.8 nm), indicating that Q10 pretreatment may have affected the size of the exosomes (Figure 2c). To track internalization of the exosomes in the HaCaTs, we incubated the cells with PKH67-labled Exos and Q10-Exo. As shown in the laser scanning confocal microscopy images in Figure 2d, the HaCaTs emitted strong fluorescence signals of PKH67, which confirmed internalization of the exosomes.

### Q10-Exo restored the viability and proliferation of HaCaTs under hyperglycemic conditions

**Figure 3:**
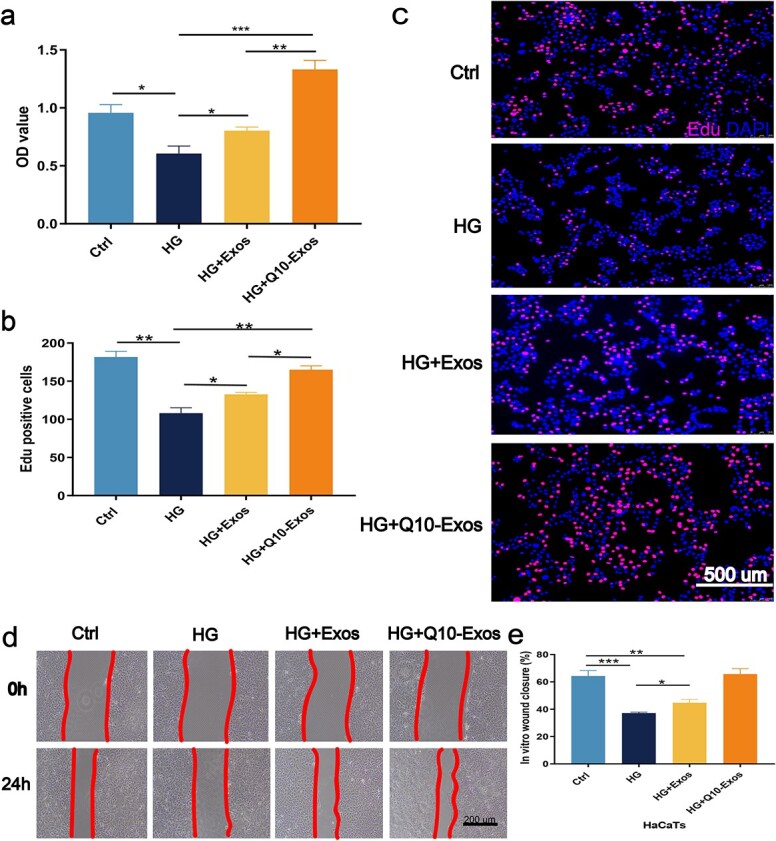
Q10-Exo protected against HG-induced inhibition of HaCaTs viability and cell proliferation. (**a**) CCK-8 assay was performed to assess the Q10-Exo effect on HaCaTs viability. (**b**,**c**) EdU incorporation assay was performed to evaluate the effect of Q10-Exo on HaCaTs proliferation. Scale bar: 500 μm. (**d**) Representative images of *in vitro* wound-healing assays to evaluate the effect of Q10-Exo on HaCaTs migration. Scale bar: 200 μm. (**e**) Quantification result of scratch wound healing assays.^*^p < 0.05, ^**^p< 0.01,^***^p < 0.001. *Exos* exosome, *MDA* malondialdehyde, *GSH* glutathione, *GSSG* glutathione disulfide

As shown in Figure 3a, exosomes derived from the untreated and Q10-treated huMSCs increased the viability of the HaCaT cells under hyperglycemic conditions, and Q10-Exo demonstrated a signficantly stronger protective effect. The proliferation of HaCaT cells in HG conditions was further assessed by the 5-ethynyl-2′ -deoxyuridine (EdU) incorporation assay. The number of EdU-positive cells was higher in the Ctrl-Exo and Q10-Exo groups compared to the untreated controls, and the highest number of prolierating cells was detected in the Q10-Exo group (Figure 3b and c). Furthermore, we also analyzed the effect of Q10-Exo on the migration capacity of HaCaT cells with the scratch wound healing assay. As shown in Figure 3d, HG conditions inhibited keratinocyte migration and decreased wound coverage *in vitro*, while treatment with Ctrl-Exo and Q10-Exo rescued wound healing. Q10-Exo had a stronger impact on *in vitro* wound healing (Figure 3d). Taken together, Q10-Exo protected keratinocytes against HG-induced toxicity and restored their viability, proliferation, and migration.

### Q10-Exo attenuated ferroptosis in HaCaT cells under hyperglycemic conditions

**Figure 4:**
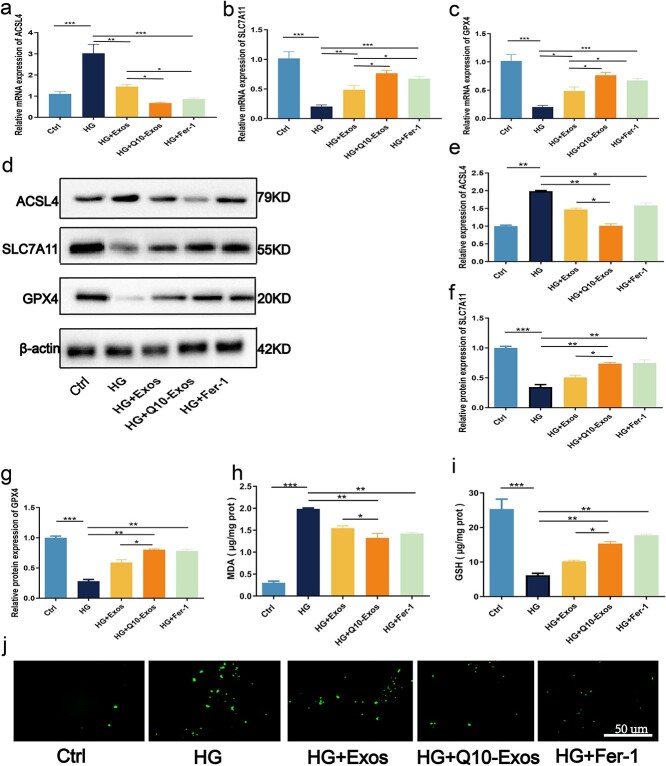
Q10-Exo protected against HG-induced ferroptosis of HaCaTs. (**a**–**c**) Effects of Q10-Exo on the mRNA expression levels of the ferroptosis genes in HaCaT keratinocytes under HG conditions. (**d**–**g**) Expression levels of ferroptosis indicators such as ACSL4, SLC7A11, and GPX4 were analyzed by western blot. (**h**) Relative MDA content was measured by the MDA detection kit in HG-exposed and normal HaCaTs cells. (**i**) GSH contents and the ratio of GSH/GSSG were measured in HG-exposed and control HaCaTs cells. (**j**) Immunofluorescence was used to detect the expression level of Fe^2+^ ions under different treatment conditions. Scale bar: 50 μm. *p < 0.05, ^**^p < 0.01,^***^p < 0.001

HaCaT cells cultured under hyperglycemic conditions showed a signficant decrease in the levels of GSH, SLC7A11, and GPX4 (Figure 4b,c,d,g and i). In contrast, HG exposure significantly increased the levels of ACSL4 mRNA and protein, MDA, and Fe^2+^(Figure 4a,d,e,h and j). Treatment with both Ctrl-Exo and Q10-Exo attenuated ferroptosis under HG conditions, and decreased MDA and Fe^2+^ levels. However, Q10-Exo further enhanced the antioxidant capacity of the HaCaT cells and significantly reduced the levels of ferroptosis markers. Furthermore, Q10-Exo also decreased the rate of cell death to a greater extent compared to Ctrl-Exo, suggesting that Q10-Exo had a more substantial inhibitory effect on ferroptosis.

### miR-548ai and miR-606 are the key bioactive molecules of Q10-Exo

**Figure 5:**
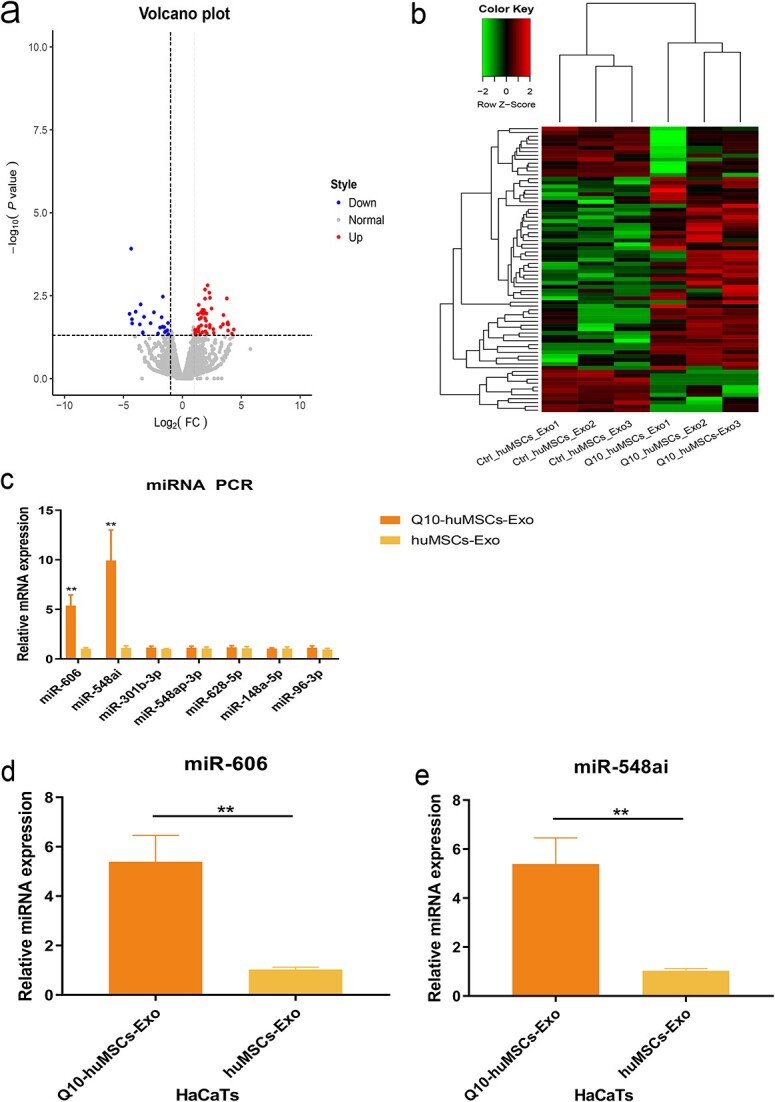
Expression profiles of miRNA in Q10 pretreated-derived extracellular vesicles (EVs). (**a**) Volcano plot exhibits the difference in miRNA expression content of huMSC-secreted exosomes after Q10 pretreatment. (**b**) Heat map shows differential expression of miRNAs between Q10-derived EVs isolated from standard control. (**c**) qPCR validated the expression level of selected miRNAs with the top seven sequencing results in Q10-huMSC exosomes. (**d**,**e**) Expression level of miR606 and miR-548ai, respectively, in HaCaTs cultured with Q10-huMSCs-Exo or huMSCs-Exo. ^**^p < 0.01. *huMSCs* umbilical cord mesenchymal stem cells

Given that exosomes exert their therapeutic effects primarily through miRNAs, we screened for the differentially expressed miRNAs (DEmiRNAs) between the Ctrl-Exo and Q10-Exo groups, with | log2(fold-change) | > 1 and *P*-adj < 0.05 as the thresholds. The clustered heat map and volcano plot of the DEmiRNAs are shown in Figure 5a and b. Based on the above screening criteria, 45 miRNAs were upregulated and 20 miRNAs were downregulated in the Q10-Exo relative to the Ctrl-Exo group (Figure 5a and b). We validated the top seven DEmiRNAs through real-time quantitative polymerase chain reaction (RT-qPCR) and found that miR-606 and miR548ai were significantly upregulated in Q10-Exo, whereas the others did not exhibit a significant change in expression (Figure 5c). Consistent with the above results, miR-606 and miR548ai were also upregulated in HaCaT cells after treatment with Q10-Exo (Figure 5d). Altogether, these findings suggest that miR-606 and miR-548ai are critical exosomal cargoes secreted by Q10-treated huMSCs, which can be effectively internalized by HaCaT cells.

### miR-548ai and miR-606 promoted the migration and proliferation of HaCaT cells *in vitro*

**Figure 6:**
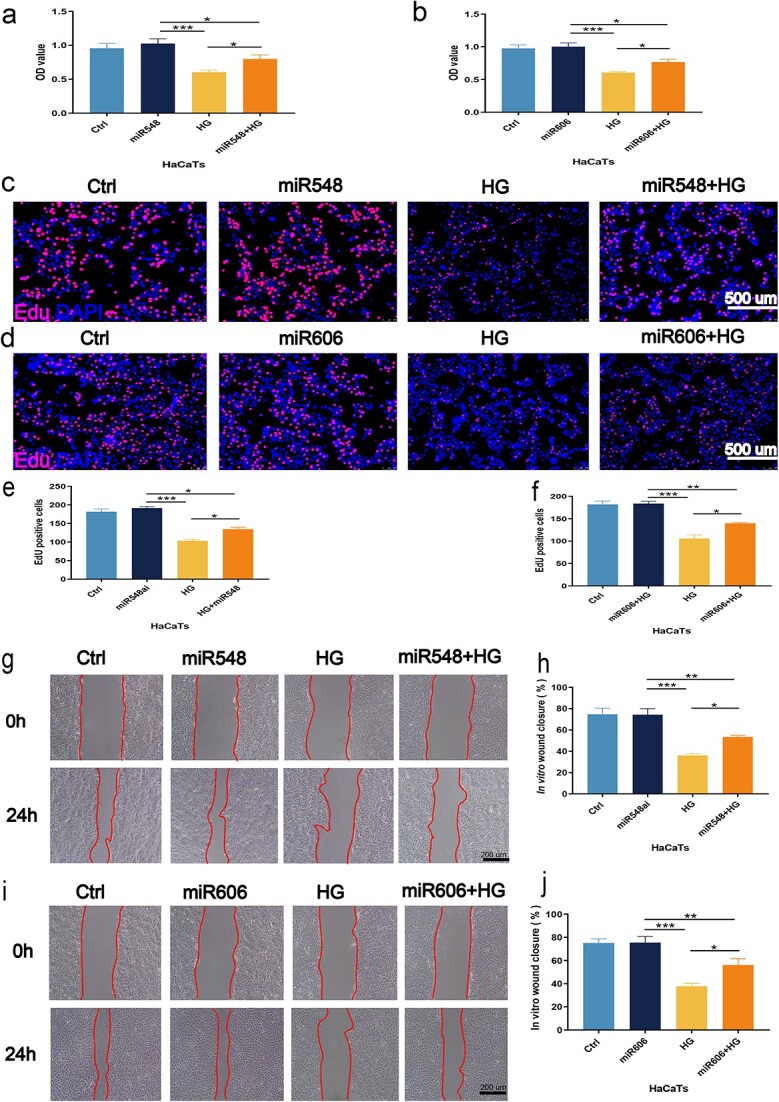
Pro-migration and proliferation effect of miR-548ai and miR-606 on HaCaT cells. (**a**,**b**) CCK-8 assay was performed to assess the Q10-Exo effect on HaCaTs viability following miR-548ai or miR-606 overexpression. (**c**–**f**) EdU incorporation assay was performed to evaluate the effect of miR-548ai and miR-606 on HaCaTs proliferation. Scale bar: 500 μm. (**g**–**j**) *In vitro* wound healing assay was performed to evaluate the effect of miR-548ai and miR-606 overexpression on HaCaTs migration. Scale bar: 200 μm. ^*^p < 0.05, ^**^p < 0.01, and ^***^p < 0.001, respectively. *HG* high glucose, *Exos* exosome

To further verify whether miR-606 and miR-548ai play a role in the proliferation and migration of keratinocytes, we transfected HaCaT cells with miR-606 and miR-548ai mimic, and analzyed their viability, proliferation, and migration. As expected, the miR-606 and miR-548ai mimics restored the viability of the HaCaT cells (Figure 6a and b) and significantly increased the number of EdU-positive cells (Figure 6c–f) under hyperglycemic condition. In addition, both miR-606 and miR-548ai rescued the migration ability of HaCaT cells in response to HG exposure (Figure 6g–j). Taken together, Q10-Exo protected keratinocytes against the effects of hyperglycemia by upregulating miR-548ai and miR-606.

**Figure 7:**
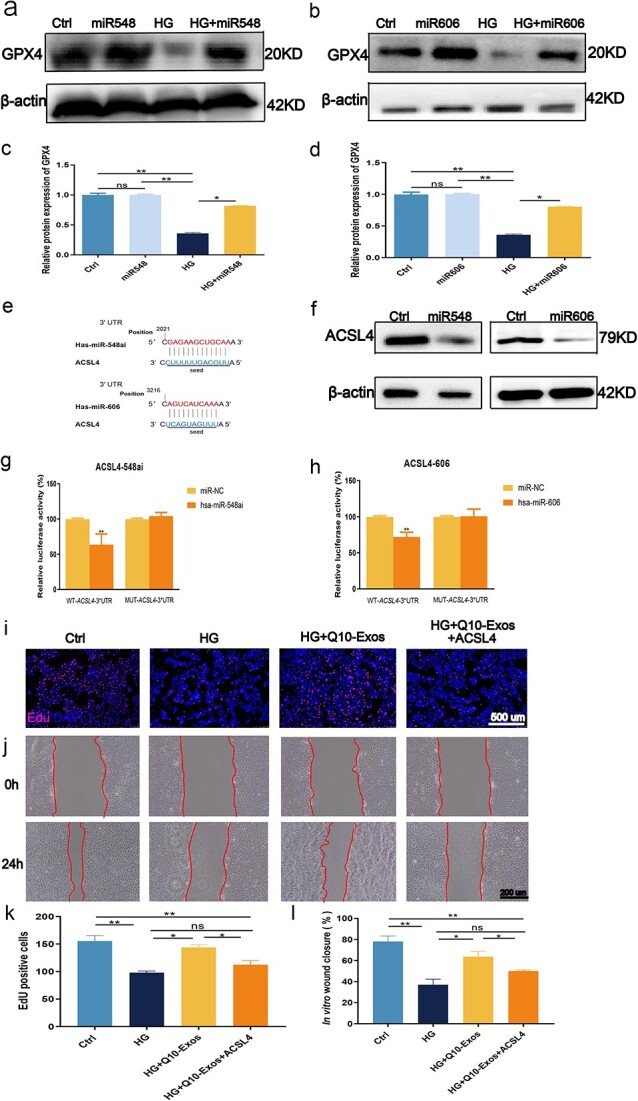
miR-548ai and miR-606 exert pro-migration and anti-ferroptosis effects by targeting ACSL4 on HaCaT cells. (**a**–**d**) Western blotting analysis for the levels of GPX4 expression in HaCaTs following miR-548ai or miR-606 overexpression. (**e**) miR-548ai and miR-606 targeting sequence in the 3′-UTR of ACSL4. (**f**) Western blotting analysis of GPX4 with β-actin as the reference. (**g**,**h**) Luciferase reporter assay was performed to confirm that ACSL4 is the target gene of miR-548ai and miR-606. (**i**) Representative images of EdU staining. Scale bar: 500 μm. (**j**) Representative images of the wound-healing scratch assays. Scale bar: 200 μm. (**k**) Statistical results of scratch wound-healing assays. (**l**) Quantitative results of EdU-labelled cells. ^*^p < 0.05 and ^**^p < 0.01; ns, not significant. *HG* high glucose, *Exos* exosome

**Figure 8:**
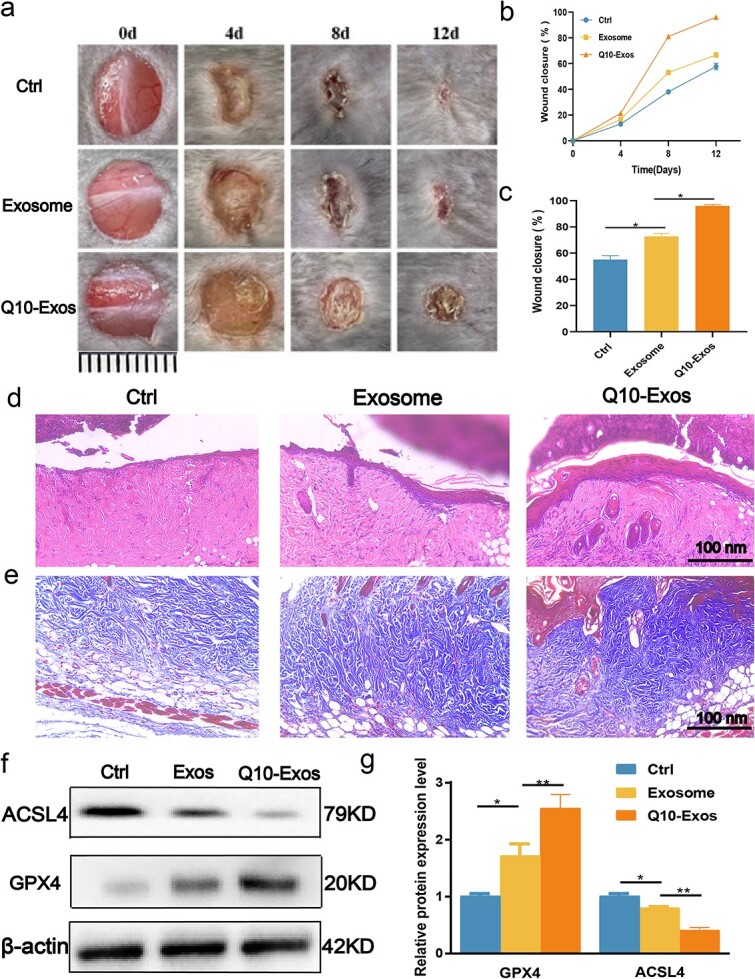
Q10-Exo transplantation promotes diabetic wound healing. (**a**) Representative images of full-thickness defects in diabetic rats receiving treatment with PBS (control), exosomes, and Q10-Exo at Days 0, 4, 8, and 12 postoperatively (scale bar: 1 cm). (**b**,**c**) Wound closure rates of the four groups (*n* = 6). (**d**) H&E staining of wound length at Day 12. Scale bar: 100 nm. (**e**) Masson trichrome staining on day 12. Scale bar: 100 nm. (**f**,**g**) Western blotting analysis for the ferroptosis-associated protein expression of ACSL4 and GPX4 in three groups (*n* = 3). ^*^p< 0.05 and ^**^p < 0.01

### miR-548ai and miR-606 inhibited ferroptosis in HaCaT cels through direct repression of ACSL4

Database gene prediction tools suggested a binding site for miR-606 and miR-548ai in ACSL4 that is highly conserved between mouse and man. Therefore, to determine whether miR-548ai and miR-606 directly target ACSL4, we constructed luciferase reporter plasmids containing the putative WT or mutant binding sites for miR-548ai or miR-606 respectively within the 3′-UTR of ACSL4 (Figure 7b and c, Supplementary Figure 2). Co-transfection of the luciferase reporter construct with WT binding sites and the miR-548ai or miR-606 mimic led to a signficant reduction in luciferase activity compared to that with the vector containing mutant 3′-UTR. Furthermore, overexpression of ACSL4 in the HaCaT cells (Figure 7d) reversed the upregulation of GPX4 by Q10-Exo (Figure 7e). The increased migration capacity induced by Q10-Exo was partly reversed by ACSL4 overexpression (Figure 7f and g), indicating that miR-548ai and miR-606 target ACSL4 to repress ferroptosis. In conclusion, the above results demonstrated that the Q10-Exo attenuates ferroptosis in keratinocytes by downregulating ACSL4 through the delivery of miR-548ai and miR-606.

### Q10-Exo accelerated diabetic wound healing *in vivo* by inhibiting ferroptosis

To evaluate the effect of Q10-Exo on diabetic wound healing, we induced full-thickness skin injuries in DB/DB rats, and treated the wounds with PBS, Exo, and Q10-Exo for 0, 4, 8, and 12 days. The wound area in the Exo and Q10-Exo groups were significantly smaller compared to that in the control group on day 8, and the reduction was more prominent in the Q10-Exo group (Figure 8a and b). HE staining demonstrated that the diabetic wounds in the Q10-Exo group had appropriate epithelization and healed more quickly than those in the control and Exo groups (Figure 8c). Furthermore, Masson trichrome staining demonstrated more extensive collagen deposition in the Q10-Exo group, indicating that Q10-Exo can activate Extracellular matrix (ECM) modeling (Figure 8d). Finally, Q10-Exo significantly downregulated ACSL4 and upregulated GPX4 (Figure 8e–g). Altogether, these findings suggest that Q10-Exo can accelerate diabetic wound healing by inhibiting ACSL4-induced ferroptosis.

## Discussion

Re-epithelialization of the skin is one of the critical steps in wound healing and is driven by the proliferation of keratinocytes and their migration toward the wound site [8]. Keratinocyte migration is impaired in diabetics, which results in poor re-epithelialization during wound healing. In this study, we found that hyperglycemic conditions impaired the migration of human keratinocytes *in vitro* by inducing ferroptosis. Yang *et al*. [43] have demonstrated that exosomes derived from MSCs pre-treated with Interferon gamma (IFN-γ) expressed high levels of miR-125a and miR-125b, and exhibited anti-inflammatory effects against colitis. In our study as well, pre-treatment of huMSCs with Q10 optimized the therapeutic efficacy of the exosomes. Q10-Exo enhanced the proliferation and migration of HaCaT cells under HG conditions *in vitro* and accelerated skin wound healing in diabetic mice. At the molecular level, Q10-Exo downregulated the ferroptosis indicator ACSL4 in the keratinocytes by delivering miR-548ai and miR-606.

Ferroptosis is a non-apoptotic form of cell death that was first observed in oncogenic Reticular activating system (RAS)-selective erastin-treated cancer cells that did not display the classic features of apoptosis. Some studies have reported a role of ferroptosis in the diabetic wound healing process [44]. HaCaT cells cultured under HG conditions showed decreased viability, Fe^2+^ accumulation, reduction in GSH levels, increased MDA levels, and mitochondrial disruption. Furthermore, the ferroptosis inhibitor DFO as well as Q10-Exo attenuated the decline in cellular viability due to HG conditions. These findings suggest that ferroptosis is a key mechanism underlying dysfunctional diabetic wound healing induced by hyperglycemia, and the therapeutic effects of Q10 may involve inhibition of ferroptosis. Consistent with this hypothesis, GPX4 and SLC7A11 were downregulated while ACSL4 was upregulated in diabetic skin tissues compared to normal skin tissues. ACSL4 is a pro-ferroptosis enzyme that catalyzes the esterification of Coenzyme A (CoA) to free fatty acids in an ATP-dependent manner. Specifically, long-chain polyunsaturated fatty acids, such as arachidonic acid and adrenic acid, are preferentially involved in lipid peroxidation. Thus, ACSL4 is an essential biomarker indicating ferroptosis [39].

Xiong *et al*. recently engineered exosomes with pro-migration, anti-inflammatory, and reactive oxygen species-scavenging functions for diabetic wound healing [45]. Furthermore, huMSC-derived exosomes can epigenetically regulate multiple cellular processes through miRNAs [23]. Exosomal miRNAs are more stable than their parental counterparts and can resist degradation by circulating nulceases due to the lipid bilayer of the exosomes [21]. However, only a few miRNAs have been identified so far that regulate ferroptosis during diabetic wound healing. To investigate the potential mechanism of the inhibitory effect of Q10-Exo on ferroptosis, we analyzed the differentially expressed miRNAs in the exosomes derived from untreated and Q10-treated huMSCs, and found that miR-606 and miR-548ai were particularly abundant in Q10-Exo. Furthermore, both miRNAs promoted keratinocyte proliferation and migration *in vitro* and inhibited ferroptosis. Therefore, we surmised that the stronger protective effect of Q10-Exo against ferroptosis compared to that of Ctrl-Exo can be attributed to the enrichment of miR-606 and miR-548ai in the former, especially since Q10 is known to regulate the expression of multiple miRNAs and miRNA processing enzymes [46]. Based on database prediction, we identified ACSL4 as a potential target of miR-606 and miR-548ai. miRNAs regulate gene expression by degrading or inhibiting the translation of mRNAs by targeting the 3′ UTR. Both miR-606 and miR-548ai mimics significantly reduced the expression of ACSL4 in the keratinocytes, and the luciferase reporter assay confirmed the possibility of each miRNA binding to the 3′ UTR of ACSL4.

Song *et al*. [47] have previously shown that HuMSC-derived exosomes inhibited ferroptosis in cardiomyocytes after myocardial infarction by repressing the Fe^2+^ transporter DMT1 through miR-23a-3p. In our study, Q10-Exo also accelerated wound healing in the DB/DB mice compared to Ctrl-Exo, and significantly inhibited ferroptosis in the diabetic skin tissues, which confirmed the bioinformatic prediction. In addition, Q10-Exo significantly increased the level of GPX4 protein and inhibited ACSL4, which is consistent with the delivery of miR-606 and miR-548ai.

Nevertheless, our study has some limitations. For example, the potential effects of the specific exosomal miRNAs on ferroptosis need further investigation. Moreover, ACSL4 may not be the only regulator of ferroptosis in keratinocytes. We were also unable to elucidate the role of Q10-Exo in diabetes-induced inflammation. Furthermore, since only the top seven upregulated miRNAs were selected to study the therapeutic mechanism of Q10-Exo, the role of the remaining differentially expressed miRNAs cannot be excluded. Finally, the doses and timing of Q10-Exo injections have to be optimized for future clinical applications.

## Conclusions

This study describes for the first time that Q10-Exo significantly improved keratinocyte viability and inhibited the ferroptosis process *in vitro*. Q10-Exo promoted the proliferation and migration of keratinocytes, and inhibited ferroptosis under hyperglycemic conditions by delivering miR-548ai and miR-660. Q10-Exo also enhanced cutaneous wound healing in diabetic mice by repressing ACSL4-mediated ferroptosis.

## Abbreviations

ACSL4: Acyl-coenzyme A synthase long-chain family member 4; BMSCs: Bone marrow-derived stem cells; Ctrl-Exo: Exosomes isolated from the control [2]; DEmiRNA: Differentially expressed miRNAs; DFO: Desferrioxamine; EdU: 5-Ethynyl-2′ -deoxyuridine; GPX4: Glutathione peroxidase; GSH: Glutathione; GSSG: Glutathione disulfide; HaCaT: Human keratinocytes; HE: Hematoxylin and eosin; HG: High glucose; huMSCs: Umbilical cord mesenchymal stem cells; MDA: Malondialdehyde; miRNA: MicroRNA; OD: Optical density; Q10: Coenzyme Q10; Q10-Exo: Exosomes derived from Q10-treated huMSCs; SLC7A11: Solute carrier family 7 member 11; TEM: Transmission electron microscopy; WT: Wild-type.

## Supplementary Material

Supplementary_File_tkae054

## Data Availability

Analyzed RNA-seq data regarding diabetic and acute skin human wounds have been deposited in the Gene Expression Omnibus (GEO) database under accession codes GSE97615 and GSE134431.
