# Supplementary material for: The role of Q10 engineering mesenchymal stem cell-derived exosomes in inhibiting ferroptosis for diabetic wound healing
Source: Burns Trauma. 2024 Nov 26;12:tkae054. doi: 10.1093/burnst/tkae054 (PMC11596300; doi:10.1093/burnst/tkae054)
Supplement: Supplementary_File_tkae054 [file supplementary_file_tkae054.docx]

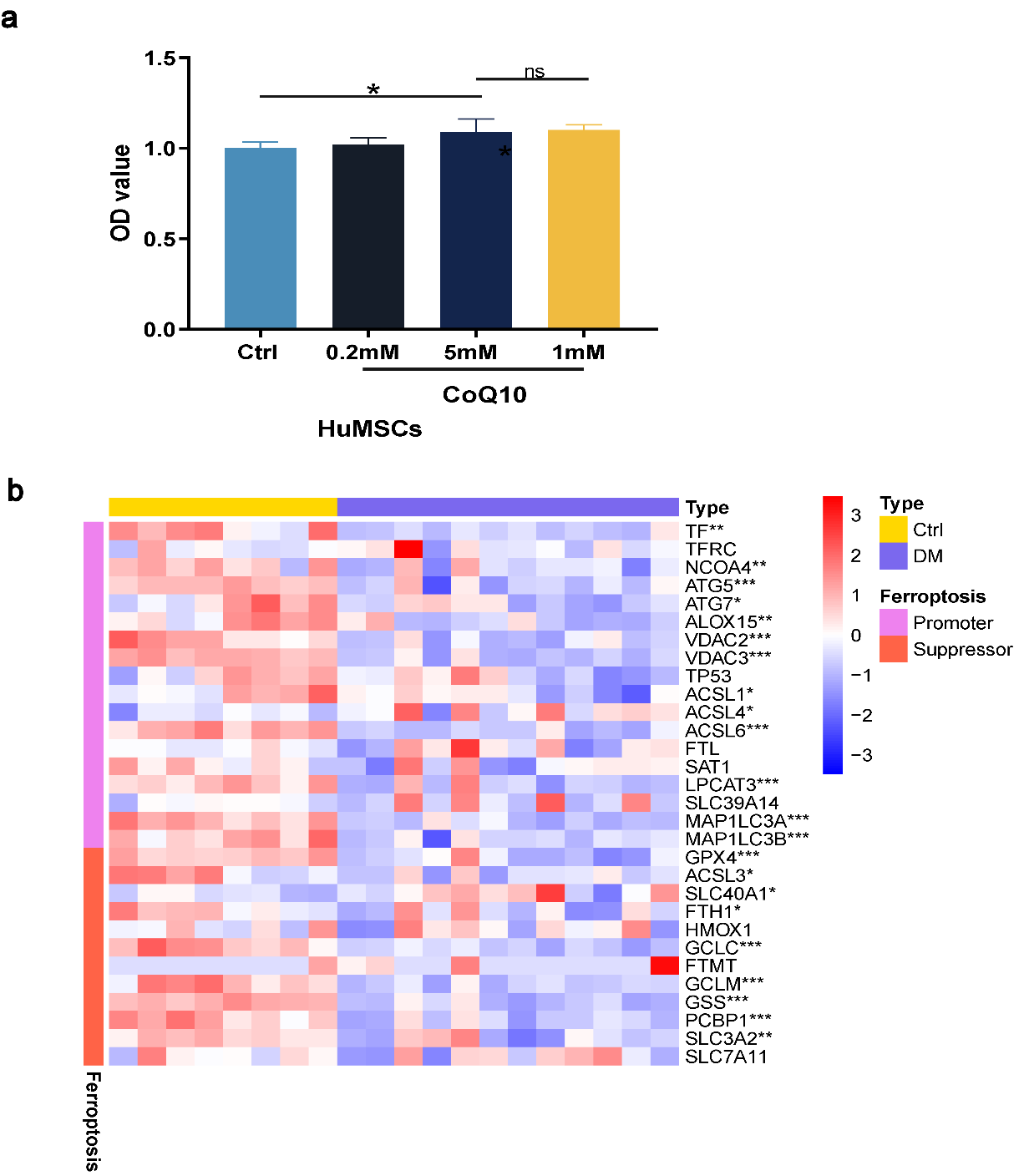


**Supplementary Figure 1:** a. Cell viability of HUMSC cells treated with various concentrations of CoQ10 through CCK‐8 assay. b. Heatmap depicting the expression pattern of ferroptosis-inducible and ferroptosis-suppressor genes in the epidermis between the control(Ctrl) and DM(Diabetics) groups.


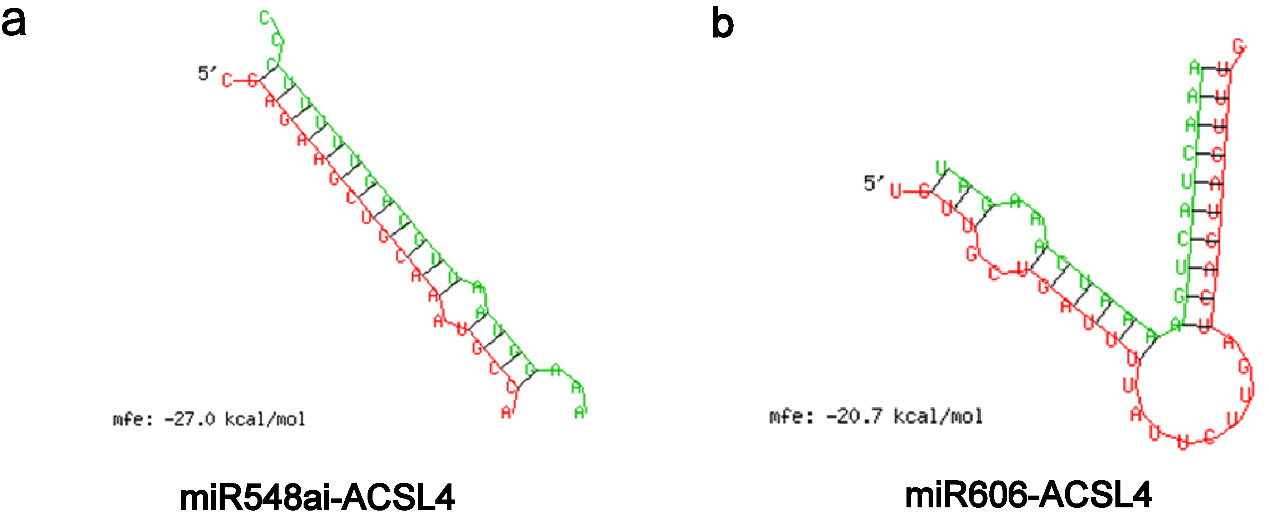


**Supplementary Figure 2:** a-b. miR548ai and miR606 binding with ACSL4 with the minimum free energy were calculated using the RNAHybrid.
